# The Ethanol Fraction of White Rose Petal Extract Abrogates Excitotoxicity-Induced Neuronal Damage *In Vivo* and *In Vitro* through Inhibition of Oxidative Stress and Proinflammation

**DOI:** 10.3390/nu10101375

**Published:** 2018-09-26

**Authors:** Jung-Min Yon, Yun-Bae Kim, Dongsun Park

**Affiliations:** 1 Veterinary Medical Center and College of Veterinary Medicine, Chungbuk National University, Cheongju, Chungbuk 28644, Korea; yonjungmin@gmail.com (J.-M.Y.); solar93@cbu.ac.kr (Y.-B.K.); 2 Department of Biology Education, Korea National University of Education, Cheongju, Chungbuk 28173, Korea

**Keywords:** white rose petal extract, kainic acid, excitotoxicity, neuroprotection, antioxidation, anti-inflammation

## Abstract

Since oxidative stress and inflammation are involved in seizure-related neurotoxicity, the neuroprotective effect of a white rose (*Rosa hybrida*) petal extract (WRPE) in mice that are challenged with kainic acid (KA) were examined using behavioral epileptiform seizures as well as biochemical and morphological parameters of oxidative stress and inflammation. WRPE (50–200 mg/kg) was orally administered to male ICR mice for 15 days, and intraperitoneally challenged with KA (30 mg/kg). Seizure activity, lipid peroxidation, inflammatory cytokines, and related enzymes were analyzed in the brain tissue, in addition to the morphological alterations in the hippocampal pyramidal neurons. Separately, antioxidant ingredients in WRPE were analyzed, and antioxidant, anti-inflammatory, and neuroprotective activities of WRPE were investigated in HB1.F3 human neural stem cells (NSCs) to elucidate underlying mechanisms. Total polyphenol and flavonoid contents in WRPE were 303.3 ± 15.3 mg gallic acid equivalent/g extract and 18.5 ± 2.2 mg catechin/g extract, respectively. WRPE exhibited strong radical-scavenging activities and inhibited lipid peroxidation in vitro, and protected glutamate-induced cytotoxicity in NSCs by suppressing inflammatory process. Treatment with WRPE attenuated epileptiform seizure scores to a half level in KA-challenged mice, and decreased hippocampal pyramidal neuronal injury and loss (cresyl violet and DAPI staining) as well as astrocyte activation (GFAP immunostaining). Lipid peroxidation was inhibited, and mRNA expression of antioxidant enzymes (GPx, PHGPx, SOD1, and SOD2) were recovered in the brain tissues. Inflammatory parameters (cytokines and enzymes) including NF-kB, IL-1β, TNF-α, IL-6, HMGB1, TGF-β, iNOS, COX2, and GFAP mRNAs and proteins were also down-regulated by WRPE treatment. Taken together, the results indicate that WRPE could attenuate KA-induced brain injury through antioxidative and anti-inflammatory activities.

## 1. Introduction

Seizures induce neuronal death through over-activation of glutamate receptors [1], which might be mediated by excessive calcium ion influx into neurons and related enzymatic as well as non-enzymatic neuronal injuries [2]. Glutamate receptor-mediated excitotoxicity is well known to be a key causative factor in diverse neurological diseases [3].

Over the past 10 years, several studies have confirmed that inflammatory processes in the brain might constitute a crucial mechanism of seizures and epilepsy [4,5,6]. For example, steroid and other anti-inflammatory treatments display anti-convulsant activity in some pharmaco-resistant epileptic patients [6]. Additionally, the level of proinflammatory cytokines has been observed to increase in febrile seizures [4]. Particularly, the increased level of cytokines such as interleukin-6 (IL-6) [7] and IL-1β [8] in cerebrospinal fluid from epilepsy patients and the aggravating effect of IL-1β, one of the most important proinflammatory cytokines in experimental models of epilepsy [9], suggest that the studies on inflammatory factors that are involved in epileptogenesis can bring new insights into the molecular mechanisms of epilepsy.

Kainic acid (KA), an analogue of glutamate, has been used as a model compound for the study of neurotoxicity of various excitatory amino acids (EAAs), because KA triggers temporal lobe epileptiform seizures that are known to be associated with the excessive release of glutamate that may underlie the pathogenesis of neuronal injury [10,11,12]. Systemic administration of KA induces neuronal damage by over-activating glutamate receptors, resulting in necrosis and apoptosis of intracellular organelles following intracellular calcium overload [1]. Moreover, KA mediates the enhancement of inflammatory cytokines such as IL-1β and tumor necrosis factor-α (TNF-α) and the generation of oxidative stress in the brain [13]. KA may induce neuronal damage through the excessive production of reactive oxygen species (ROS) and lipid peroxidation [14,15], as triggered by the activation of EAA receptors.

Although various antiepileptic drugs have been prescribed to prevent seizures, interfere with epileptogenesis, and eliminate neurodegeneration in most patients, the development of novel natural products is important for patients who experience drug resistance and deleterious side effects to traditional drugs [16]. Thus, many studies have focused on natural products possessing anti-epileptogenic, anti-inflammatory, and antioxidative activities. Earlier, we demonstrated that an extract of rough aster exhibited a neuroprotective effect via antioxidative activity in mice that are challenged with KA [17]. Vineatrol, a grape-derived polyphenolic fraction that is isolated from vine-shoot extracts, and curcumin, a dietary antioxidant, also protected against KA-induced seizures via an antioxidative effect [18,19]. Resveratrol, well known with its anti-inflammatory, anti-cancerous, and neuroprotective effects, has been shown to have antiepileptic activity in KA-injected animals [20]. Additionally, capsaicin prevented epileptogenesis through its anti-ictogenic, hypothermic, antioxidative, and anti-inflammatory effects [13].

Previously, we reported that white rose petal extract (WRPE) displayed a broad spectrum of antioxidant [21], anti-bacterial and anti-fungal [22], anti-allergic, and anti-inflammatory properties [23]. Notably, a butanol fraction of WRPE containing high amounts of gallic acid and volatile compounds reduced the infarction volume in an animal model of ischemic cerebral stroke [24]. Such results led us to investigate the neuroprotective effects of WRPE in KA-induced temporal lobe epilepsy model by analyzing the neurobehavioral and morphological effectiveness in the mouse brain, in addition to antioxidative, anti-inflammatory, and neuroprotective potentials of WRPE in vitro and in vivo as underlying mechanisms.

## 2. Materials and Methods

### 2.1. Preparation of the WRPE

Fresh white flowers of *Rosa hybrida* Colorado were procured from a rose farm (Rose Rangs, Jincheon, Korea). The flowers were collected in May 2014, completely dried in the shade to obtain dried petals, and then ground in a rotor mill (Laval Lab Inc., Laval, QC, Canada). The dried petal powder was extracted in an ultrasonic bath with 50% ethyl alcohol three times at 60 °C, and then filtered to obtain WRPE. The resulting fractions were completely dried in a vacuum evaporator.

### 2.2. HPLC Analysis of Free Phenolic Acids

High-performance liquid chromatographic (HPLC) analysis was performed while using a slightly modified protocol of Kim et al. [25]. WRPE was extracted with an ethyl acetate and ether mixture (1:1 *v*/*v*) using a separatory funnel. The upper phase was evaporated using a rotatory evaporator at 40 °C (N-1000, Eyela, Tokyo, Japan), dissolved in methanol, and filtered through a 0.2-μm syringe filter (Millipore, Billerica, MA, USA). An HPLC series system (ACME 9000 system, Younglin, Anyang, Korea) with a UV detector was used, and separation was achieved using a Mightysil RP-18 GP column (4.6 × 250 mm, 5 μm, Kanto Chemical, Tokyo, Japan). The absorbance was measured at 280 nm. The mobile phases were 0.1% acetic acid in acetonitrile (solvent A) and 0.1% acetic acid in water (solvent B). The injection volume was 20 μL and the gradient was as described in Table 1. The run time was 70 min at a flow rate of 1 mL/min.

### 2.3. Determination of Polyphenol and Flavonoid Contents

In order to measure total phenolic compounds, the sample (100 μL) was mixed with 2% Na_2_CO_3_ (2 mL). At least 3 min later, 50% Folin-Ciocalteu reagent (100 mL) was added, and the absorbance was measured at 750 nm while using a spectrophotometer (UV-1650, Shimadzu Corporation, Kyoto, Japan). The amount of phenolic ingredients was quantified using a calibration curve prepared with standard gallic acid solutions (0.1–0.5 mg/mL).

To determine total flavonoid content, distilled water (DW, 1 mL) was added to the sample (250 μL) and mixed with 5% NaNO_2_ (75 μL). Five min later, 10% AlCl_3_ (150 μL) was added. After 6 min, 1 M NaOH (500 μL) was added, and the absorbance was measured at 510 nm using the spectrophotometer. A calibration curve was made using (+)-catechin hydrate solutions (0.1–0.5 mg/mL) under the same procedures.

### 2.4. Measurement of Antioxidant Activity

Antioxidant activities of WRPE were measured with radical-scavenging and lipid peroxidation assays in vitro. 2,2′-azino-bis-3-ethylbenzthiazoline-6-sulphonic acid (ABTS) and 1,1-diphenyl-2-picrylhydrazyl (DPPH) radical-scavenging activity and determining the formation of thiobarbituric acid (TBA)-reactive substances (TBARS) were measured of WRPE as previously described [12]. All samples were analyzed in triplicate.

Lipid peroxidation was measured by determining the formation of thiobarbituric acid (TBA)-reactive substances (TBARS) as previously described [12].

### 2.5. Human Neural Stem Cell (NSC) Culture

The HB1.F3 human NSCs [26] were cultivated in a high-glucose Dulbecco’s Modified Eagle’s Medium (DMEM) (Biowest, Nuaillé, Cholet, France) supplemented with 10% fetal bovine serum (FBS) (Biowest, Nuaillé, Cholet, France), 100 U/mL penicillin, and 100 µg/mL streptomycin (Invitrogen, Carlsbad, CA, USA). Cultures were maintained under 5% CO_2_ at 37 °C in tissue culture flasks. The cells were grown to 90% confluency and subjected to the cytotoxicity test.

### 2.6. Lactate Dehydrogenase (LDH) Assay in NSCs

Cytotoxicity of glutamate and protective effect of WRPE were quantified by measuring LDH release from HB1.F3 NSCs. To determine median lethal concentration (50% LDH release), HB1.F3 cells (1 × 10^6^ cells) were treated with various concentrations (0–50 mM) of sodium glutamate for 2 h. After washing with DMEM medium, the cell viability was determined 24 h later while using the LDH assay. The LDH content was determined using a commercial non-radioactive LDH assay kit (Promega, Madison, WI, USA) according to its protocol. In order to evaluate the protective effect of WRPE, HB1.F3 cells were treated with various concentrations (0–1250 µg/mL) of WRPE and, 30 min later, with 2.5 mM glutamate. After washing with fresh medium in 2 h, the cells were cultivated for 24 h further to measure LDH release. The experiments were performed in triplicate.

### 2.7. Quantitative Real-Time PCR in NSCs

Total RNA was isolated from HB1.F3 cells using TRIzol Reagent (Invitrogen, CA, USA) according to the manufacturer’s instructions. Quantitative real-time PCR were measured according to previously described methods [27]. Glyceraldehyde 3-phosphate dehydrogenase (GAPDH) was used as an internal standard to normalize the expression of the target transcripts. The primer sets were used to amplify nuclear factor-κB (NF-κB), TNF-α, IL-6, inducible nitric oxide synthase (iNOS), cyclooxygenase 2 (COX2), glutathione peroxidase 1 (GPx1), phospholipid hydroperoxide GPx (PHGPx), superoxide dismutase 1 (SOD1), and SOD2 (Table 2). Triplicate data were analyzed by six independent assays using a comparative *C*t method [27].

### 2.8. Western Blot Analysis in NSC

NSCs were homogenized in 10 volumes of RIPA buffer (Thermo Scientific, Waltham, MA, USA) containing protease inhibitors (Sigma-Aldrich, St. Louis, MO, USA) and phosphatase inhibitors (Sigma-Aldrich, St. Louis, MO, USA). Western blot analysis was conducted according to previously described methods [24]. The membranes were then immunoblotted with primary antibodies, followed by incubation with horseradish peroxidase-conjugated anti-rabbit or anti-goat secondary antibodies (Santa Cruz Biotechnology, Santa Cruz, CA, USA). The antibodies used in this study are summarized in Table 3. The band densities were measured using ImageJ (NIH, Bethesda, Maryland, USA) and normalized to the density of GAPDH.

### 2.9. Animals and Treatment

Seven-week-old male ICR mice were purchased from Daehan Biolink (Eumseong, Korea). They were housed in an environmentally-controlled room with constant temperature (23 ± 3 °C), relative humidity (50 ± 10%), and 12-h light/dark cycle. The animals were fed a standard rodent chow and purified water *ad libitum*.

After acclimation to the laboratory environment for one week, the mice (*n* = 15/group) were assigned to treatment groups. WRPE at 50, 100 or 200 mg/kg/day or its vehicle (purified water) was orally administered for 15 days, and KA was injected into the intraperitoneal cavity to induce epilepsy 30 min after the last WRPE treatment. Nine animals from each group were sacrificed 4 h after KA injection to analyze the oxidative and inflammatory reactions in the brain. The remained animals (*n* = 6/group) were sacrificed three days later for the analysis of histopathological alterations. All of the experimental procedures were carried out in accordance with the Standard Operating Procedures of the Laboratory Animal Center, Chungbuk National University (CBNU), Korea, and this protocol was approved by the Institutional Animal Care and Use Committee of CBNU (Approval #: CBNUA-469-13-02).

### 2.10. Scoring of KA-Induced Seizures

Following KA and/or WRPE administration, behavioral seizure activity was rated every 10 min for a total period of 120 min according to the scales devised by Racine [28]: facial clonus (stage 1), nodding (stage 2), forelimb clonus (stage 3), forelimb clonus with rearing (stage 4), and rearing, jumping, and falling (stage 5). Animals were scored after three consecutive seizures at each stage.

### 2.11. Analysis of Cytokines and Lipid Peroxidation in the Brain Tissue

The concentration of IL-1β and TNF-α in the brain tissue was determined in the prepared supernatant with an ELISA kit (RayBiotech, Norcross, GA, USA) according to the manufacturer’s instructions. The protein concentration of the supernatants was analyzed using a Pierce™ BCA Protein Assay kit (Thermo Fisher Scientific, MA, USA). All of the samples were analyzed in duplicate and the data were expressed as pg/mg protein.

Lipid peroxidation in the brain homogenate was performed described above.

### 2.12. Quantitative Real-Time PCR in the Brain Tissue

Quantitative PCR in total RNA isolated from the brain homogenate was performed described above. The primer sets were used to amplify NF-κB, TNF-α, IL-6, iNOS, COX2, and SOD2 (Table 2).

### 2.13. Western Blot Analysis in the Brain Tissue

Western blot analysis in the brain homogenate was performed described above.

### 2.14. Nissl Staining of the Brain Tissue

In order to observe morphological alterations, brain tissues were removed and post-fixed overnight, followed by cryoprotection in a 30% sucrose solution for 48 h. Brain coronal cryosections (15 μm in thickness) including the hippocampus (−1.8 to −2.2 mm from the bregma) were obtained using a microtome (Leica, Solms, Germany) and stained with cresyl violet (Sigma-Aldrich, St. Louis, MO, USA). Neurons with normal appearance in the pyramidal cell layer of the CA3 region (mediolateral 1.5 mm, dorsoventral −1.0 mm) were counted.

### 2.15. Fluoro-Jade C (FJC) Staining of the Brain Tissue

To visualize degenerative neurons, brain sections were stained with an FJC kit (Biosensis, Thebarton, Australia), according to the manufacturer’s instructions with some modifications. In brief, after immersing in DW for 2 min, brain cryosections were incubated in potassium permanganate solution for 10 min, rinsed with DW for 2 min, and incubated in FJC solution for 10 min. The slides were then washed and mounted on coverslips with Vectashield mounting medium (Vector, Vector Laboratories, CA, USA). All of the sections were observed and photographed under a fluorescence microscope with a blue excitation light (LSM710, Zeiss, Thornwood, NY, USA).

### 2.16. Glial Fibrillary Acidic Protein (GFAP) Immunostaining of the Brain Tissue

For immunohistochemical staining of astrocytic GFAP, brain cryosections were rinsed in TBS and treated with 3% hydrogen peroxide for 5 min to block endogenous peroxidase activity. After washing with TBS and blocking with 5% BSA, the sections were incubated overnight at 4 °C with an antibody specific to GFAP (1:1000; rabbit polyclonal, Chemicon, Temecula, CA, USA) and with a secondary antibody being conjugated with Alexa Fluor-594 (1:1000; Molecular Probes, Eugene, OR, USA). The sections were counterstained with 4′,6-diamino-2-phenylindole (DAPI, Sigma-Aldrich, St. Louis, MO, USA) to identify cellular nuclei and viewed under a laser-scanning confocal microscope (LSM710; Zeiss, Oberkochen, Germany).

### 2.17. Statistical Analysis

Statistical comparisons between the groups were performed using one-way analysis of variance followed by a Tukey’s multiple comparison test. All analyses were conducted while using the Statistical Package for Social Sciences for Windows software, version 12.0 (SPSS Inc., Chicago, IL, USA). Statistical significance was assessed at *p* < 0.05. All data were expressed as the mean ± SD.

## 3. Results

### 3.1. Phenolic Compounds in WRPE and Antioxidant Activity In Vitro

**Figure 1:**
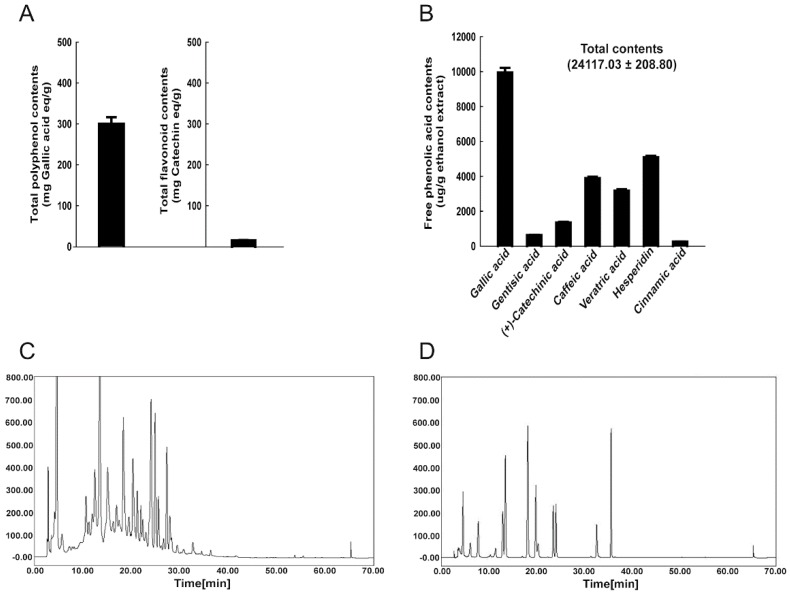
Polyphenol and flavonoid contents in white rose petal extract (WRPE). (**A**) Total amounts of phenol (gallic acid equivalent/g extract) and flavonoid (catechin equivalent/g extract) compounds. (**B**) Free phenolic acid contents in WRPE identified by HPLC. (**C**) HPLC chromatograms of WRPE. Peaks were analyzed based on HPLC chromatograms of phenolic acid standards. (**D**) HPLC chromatograms of phenolic acid standards.

The total polyphenol and flavonoid contents in WRPE were 303.3 ± 15.3 mg gallic acid equivalent/g extract and 18.5 ± 2.2 mg catechin/g extract, respectively (Figure 1A). Although we conducted quantitative analysis on 12 phenolic compounds using an HPLC (Figure 1C,D), only seven phenolic compounds (gallic acid, gentisic acid, (+)-catechinic acid, caffeic acid, veratric acid, hesperidin, and cinnamic acid) were detected in WRPE with a total amount of 24,117.0 ± 208.8 µg/g extract (Figure 1B).

**Figure 2:**
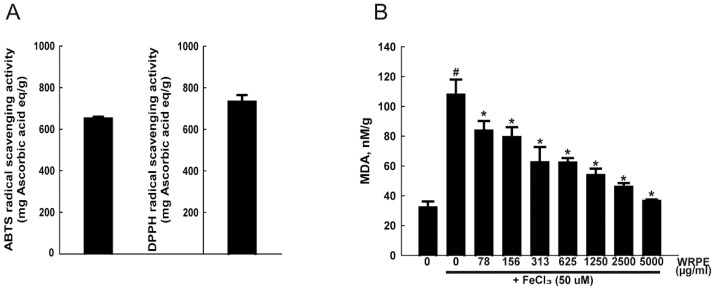
Antioxidant activities of white rose petal extract (WRPE). (**A**) 2,2′-azino-bis-3-ethylbenzthiazoline-6-sulphonic acid (ABTS) radical-scavenging activity (ascorbic acid equivalent/g extract) for 60 min. 1,1-diphenyl-2-picrylhydrazyl (DPPH) radical-scavenging activity (ascorbic acid equivalent/g extract) in 30 min. (**B**) Inhibitory activity on FeCl_3_-induced lipid peroxidation (TBARS production). *n* = 5 per treatment group. # Significantly different from normal control (*p* < 0.05). * Significantly different from vehicle control (*p* < 0.05).

In the ABTS and DPPH radical-scavenging activities, WRPE had 651.14 ± 8.42 and 732.38 ± 32.10 mg ascorbic acid equivalent/g extract, respectively (Figure 2A). There was an increase (335%) in TBARS level after exposure to FeCl_3_ (50 µM). However, treatment with various concentrations (0–5000 μg/mL) of WRPE significantly reduced the lipid peroxidation in a concentration-dependent manner (Figure 2B).

### 3.2. Neuroprotective and Anti-Inflammatory Activities of WRPE in NSCs

**Figure 3:**
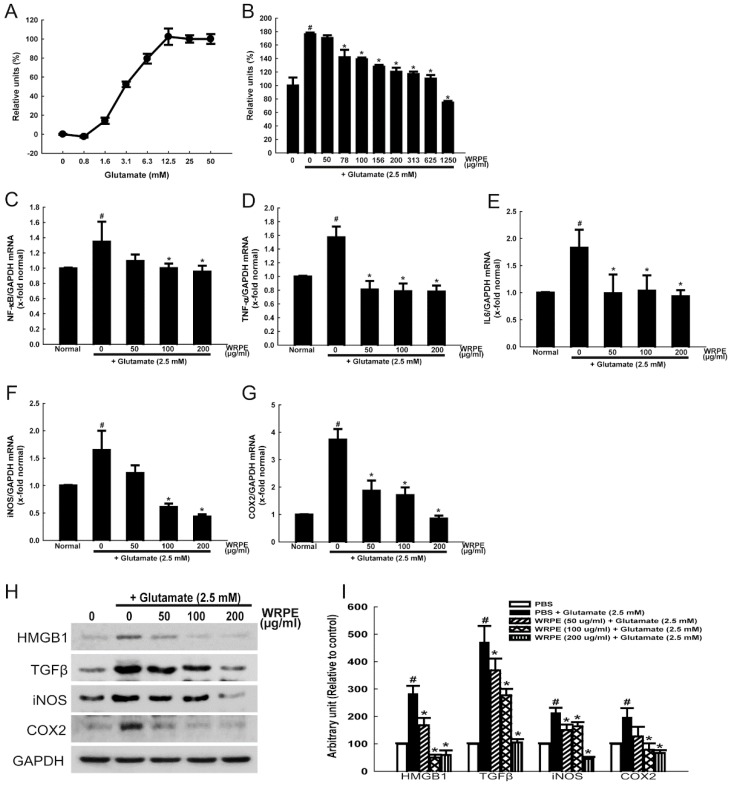
Neuroprotective activity of white rose petal extract (WRPE) against glutamate cytotoxicity in human neural stem cells (NSC). (**A**) Cytotoxicity of glutamate in LDH assay. (**B**) Protective effect of WRPE on glutamate-induced cytotoxicity in LDH assay. (**C**–**G**) Real-time PCR analysis of mRNA of NF-κB (**C**), TNF-α (**D**), IL-6 (**E**), iNOS (**F**), and COX2 (**G**) normalized to GAPDH. (**H**,**I**) Western blot analysis of proteins of HMGB1, TGF-β, iNOS, and COX2, showing representative bands (**H**) and band densities normalized to GAPDH (**I**). *n* = 5 per treatment group. # Significantly different from normal control (*p* < 0.05). * Significantly different from vehicle control (*p* < 0.05).

Following exposure to increasing concentrations (0–50 mM) of glutamate for 2 h, cell death (LDH release) increased in a concentration-dependent manner between 0.8 and 12.5 mM (Figure 3A). Higher concentrations (>12.5 mM) of glutamate did not induce further increase in LDH release, which is indicative of complete cytotoxicity at around 12.5 mM. Therefore, median (50%) lethal concentration of glutamate in NSCs was estimated to be 2.5 mM, which was used for the neuroprotective effects of WRPE in our subsequent studies.

Exposure of NSCs to 2.5 mM glutamate resulted in 176% increase in LDH release (Figure 3B). Notably, such a cytotoxicity induced by glutamate was significantly attenuated by 30 min pretreatment with WRPE (≥78 μg/mL). Although 1250 μg/mL WRPE fully inhibited the LDH release to a level lower than that in normal cells, direct cytotoxicity of WRPE was not excluded. Thus, we analyzed the anti-inflammatory effects of WRPE at doses of 50, 100, and 200 µg/mL.

Treatment of NSCs with glutamate (2.5 mM) significantly up-regulated the NF-κB mRNA expression (Figure 3C). Interestingly, however, the NF-κB expression was markedly attenuated by treatment with WRPE (50–200 μg/mL), leading to decrease to normal level at ≥100 μg/mL. In parallel with the change in NF-κB, the expression of TNF-α and IL-6 was also remarkably increased by glutamate, which was fully blocked by treatment with WRPE (50–200 μg/mL) (Figure 3D,E). According to the up-regulation of NF-κB and inflammatory cytokines, the gene expression of inflammatory enzymes, iNOS and COX2, was increased following exposure to glutamate (Figure 3F,G), which is actively involved in the regulation of proinflammatory proteins such as iNOS and COX2. Notably, pretreatment with WRPE significantly inhibited the enzyme expression in a concentration-dependent manner.

In the western blot analysis, in addition to iNOS and COX2, the production of proteins of proinflammatory cytokines such as TGF-β and high-mobility group box 1 (HMGB1) was increased by glutamate (Figure 3H,I). However, the glutamate-induced over-production of the proteins was markedly attenuated by treatment with WRPE, although there were differences in sensitivity.

### 3.3. Anticonvulsant and Antioxidant Activities of WRPE in KA-Challenged Mice

**Figure 4:**
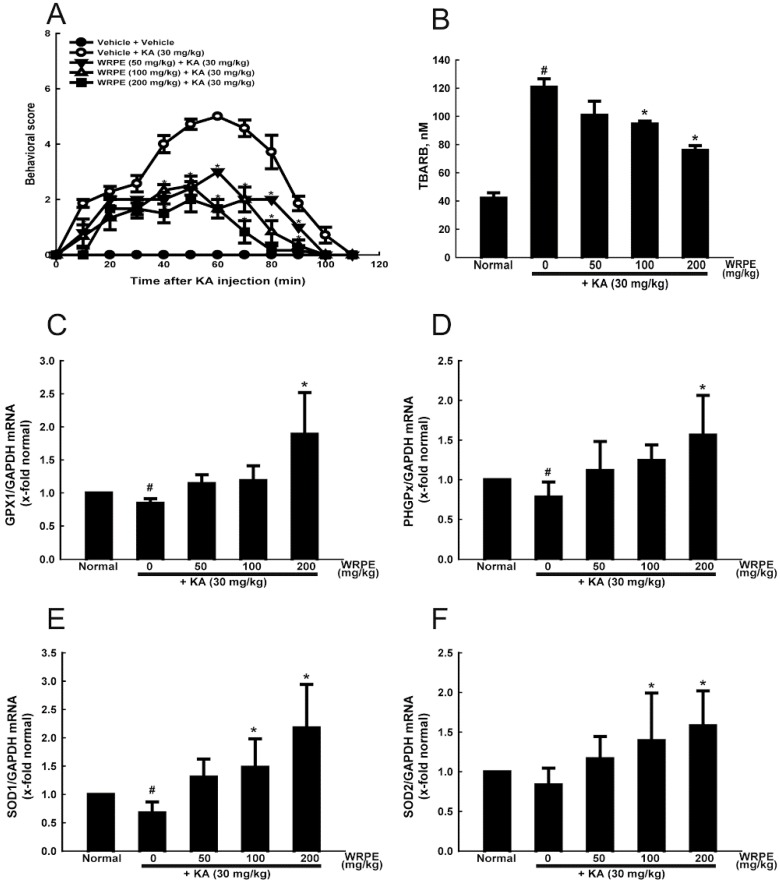
Anticonvulsant and antioxidant activities of white rose petal extract (WRPE) in kainic acid (KA)-challenged mice. (**A**) Time-course of seizure intensity following injection of KA. (**B**) Lipid peroxidation (TBARS content) in the brain tissue. (**C**–**F**) Real-time PCR analysis of mRNA of GPx (**C**), PHGPx (**D**), SOD1 (**E**), and SOD2 (**F**) normalized to GAPDH. *n* = 5 per treatment group. # Significantly different from normal control (*p* < 0.05). * Significantly different from vehicle control (*p* < 0.05).

Seizure activity increased up to 60 min after KA injection, reaching mean score of 4.78 ± 0.14, and then gradually disappeared, lasting to 110–120 min (Figure 4A). Notably, pretreatment of WRPE not only significantly attenuated the KA-induced seizure intensity, but also shortened the duration. Especially, the seizure intensity and duration were decreased to a half level and 80 min, respectively, by 200 mg/kg WRPE.

Intensive seizures that were induced by KA challenge increased the TBARS concentration up to 3 times the control level (Figure 4B). However, such lipid peroxidation was reduced by treatment with WRPE in dose-dependent manner. Such antioxidant effect of WRPE led us to investigate the relationship with antioxidant enzyme activities. Although GPx1, PHGPx, SOD1, and SOD2 mRNA levels were down-regulated by KA, WRPE treatment markedly enhanced the gene expression of all the enzymes above their control levels (Figure 4C–F).

### 3.4. Anti-Inflammatory Activity of WRPE in KA-Challenged Mice

**Figure 5:**
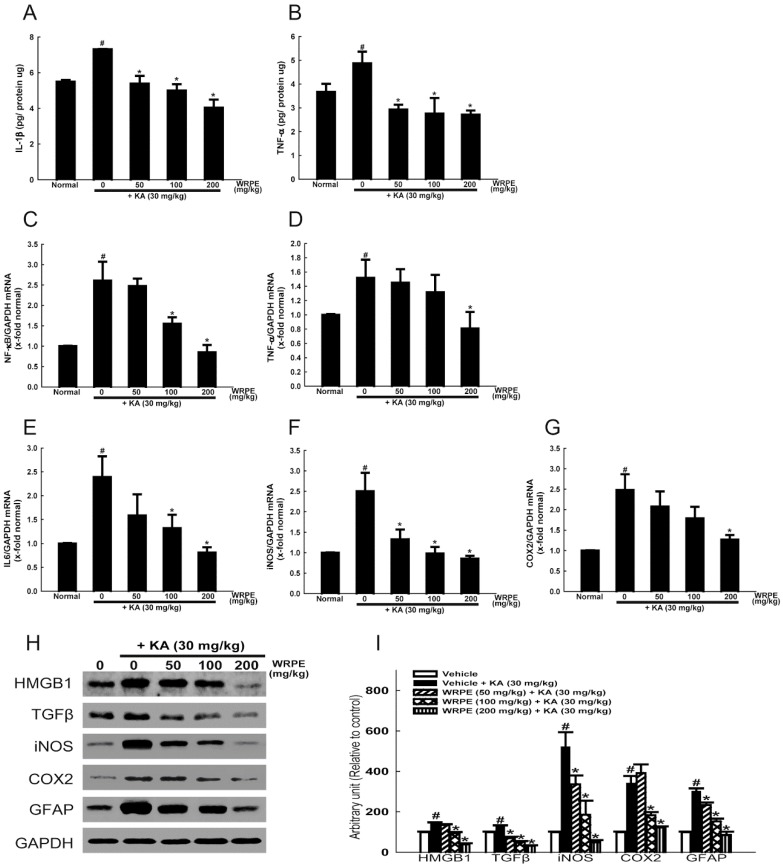
Anti-inflammatory activity of white rose petal extract (WRPE) in kainic acid (KA)-challenged mice. (**A**,**B**) ELISA analysis of IL-1β (**A**) and TNF-α (**B**) in the brain tissue. (**C**–**G**) Real-time PCR analysis of mRNA of NF-κB (**C**), TNF-α (**D**), IL-6 (**E**), iNOS (**F**), and COX2 (**G**) normalized to GAPDH. (**H**,**I**) Western blot analysis of proteins of HMGB1, TGF-β, iNOS, and COX2, showing representative bands (**H**) and band densities normalized to GAPDH (**I**). *n* = 5 per treatment group. # Significantly different from normal control (*p* < 0.05). * Significantly different from vehicle control (*p* < 0.05).

The concentration of IL-1β and TNF-α significantly increased in the brain tissue of KA-challenged mice (Figure 5A,B). As inferred from the cytokine levels, the expression of NF-κB and TNF-α mRNA was also markedly increased by KA exposure (Figure 5C,D). In addition, the mRNA expression of IL-6 as well as inflammatory enzymes iNOS and COX2 was greatly up-regulated, too (Figure 5E–G). It is of interest to note that treatment with WRPE potently lowered all of the inflammatory cytokines and enzymes, although there were differences in dose-response relationship.

In the western blot analysis, the production of proteins of inflammatory enzymes iNOS and COX2 as well as proinflammatory cytokines TGF-β and HMGB1 was markedly increased by KA-induced seizures (Figure 5H,I). Notably, however, the KA-induced over-production of the proteins was inhibited by treatment with WRPE in a dose-dependent manner.

### 3.5. Neuroprotective Effects of WRPE in KA-Challenged Mice

**Figure 6:**
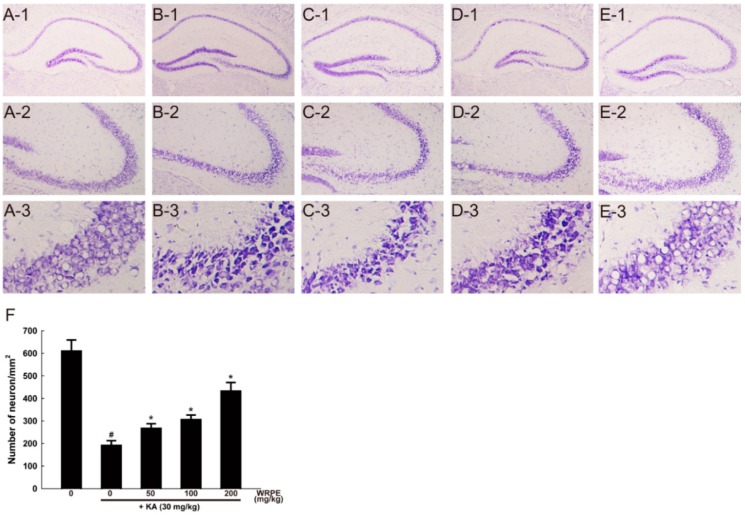
Neuroprotective activity of white rose petal extract (WRPE) in kainic acid (KA)-challenged mice observed by cresyl violet staining, showing representative microscopic findings (**A**–**E**) and the number of survived cells (**F**). (**A**) Normal (vehicle) control, (**B**) KA (30 mg/kg) alone, (**C**) KA + WRPE (50 mg/kg), (**D**) KA + WRPE (100 mg/kg), and (**E**) KA + WRPE (200 mg/kg). (**F**) Cresyl violet-positive neurons with normal appearance in the hippocampal CA3 pyramidal cell layer were counted. *n* = 3 per treatment group. # Significantly different from normal control (*p* < 0.05). * Significantly different from vehicle control (*p* < 0.05).

In cresyl violet staining, KA-induced neuronal death exhibiting many halos around shrunk cells was mainly observed in the CA3 pyramidal cell layer (Figure 6(B-1)-(B-3)). Cresyl violet-stained intact neurons in the CA3 pyramidal cell layer decreased to one-third level (Figure 6F). However, the pyramidal neurons were significantly preserved in the mouse brain pretreated with WRPE, displaying a dose-dependent effect (Figure 6C–F).

**Figure 7:**
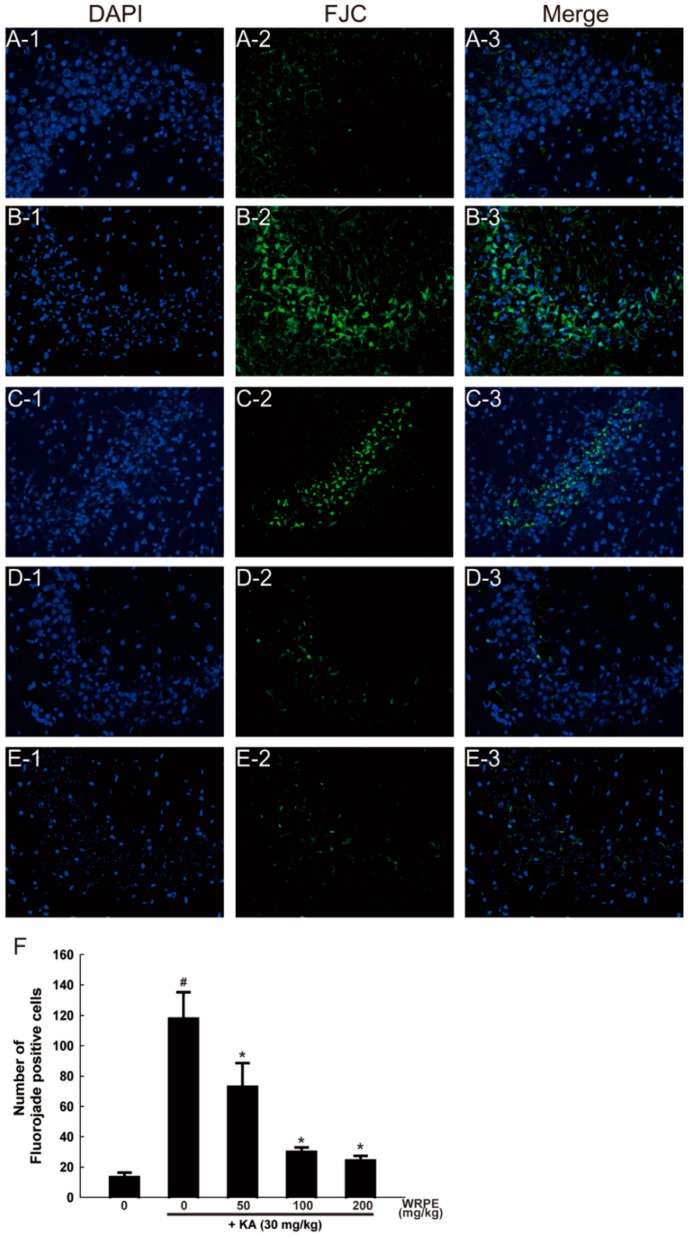
Neuroprotective activity of white rose petal extract (WRPE) in kainic acid (KA)-challenged mice observed by Fluorojade C (green-colored) staining, showing representative microscopic findings (**A**–**E**) and the number of degenerating (Fluorojade C-positive) cells (**F**). (**A**) Normal (vehicle) control, (**B**) KA (30 mg/kg) alone, (**C**) KA + WRPE (50 mg/kg), (**D**) KA + WRPE (100 mg/kg), and (**E**) KA + WRPE (200 mg/kg). (**F**) Quantification of FJC-labeled neurons in the hippocampal CA3 pyramidal cell layer. *n* = 3 per treatment group. # Significantly different from normal control (*p* < 0.05). * Significantly different from vehicle control (*p* < 0.05).

Many FJC-positive (green colored) cells were observed in the hippocampal pyramidal cell layer of KA-challenged mice (Figure 7B,F). Such degenerating neurons disappeared when WRPE was pretreated, in a dose-dependent manner (Figure 7F).

**Figure 8:**
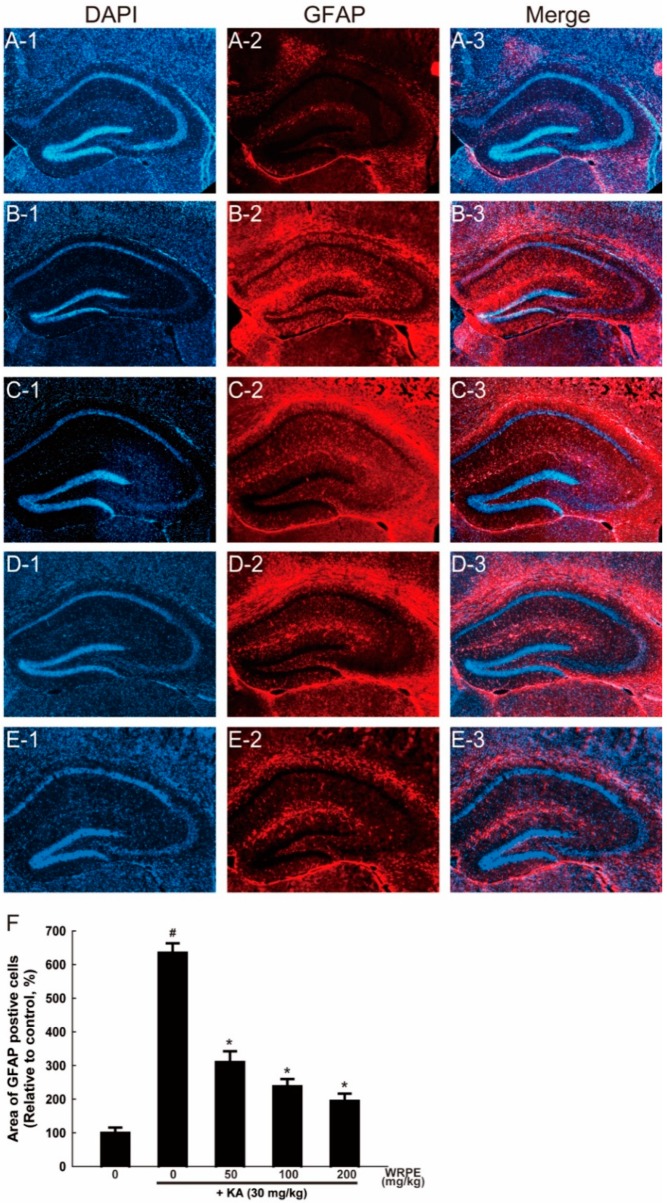
Neuroprotective activity of white rose petal extract (WRPE) in kainic acid (KA)-challenged mice observed by glial fibrillary acidic protein (GFAP) (red-colored) immunostaining, showing representative microscopic findings (**A**–**E**) and the number of activated (GFAP-positive) astrocytes (**F**). (**A**) Normal (vehicle) control, (**B**) KA (30 mg/kg) alone, (**C**) KA + WRPE (50 mg/kg), (**D**) KA + WRPE (100 mg/kg), and (**E**) KA + WRPE (200 mg/kg). (**F**) Relative quantification of GFAP-positive astrocytes in the brain coronal section. *n* = 3 per treatment group. # Significantly different from normal control (*p* < 0.05). * Significantly different from vehicle control (*p* < 0.05).

Inflammation-activated CNS astrocytes are characterized by hypertrophy and proliferation, along with an up-regulation of their cytoskeletal GFAP. There were intensive GFAP-immunoreactivities (red-colored) of activated astrocytes in the subventricular zone and striatum of the KA-challenged mice (Figure 8B,F). However, astrocytic activation was significantly inhibited by pretreatment with WRPE (100–200 mg/kg) to the normal level in the subventricular zone (Figure 8D–F). Interestingly, there were decreased DAPI-stained neurons in the CA3 pyramidal cell layer in a reversed relationship with GFAP content (Figure 8, column 1), in parallel with the neuronal death in cresyl violet staining (Figure 6). Such neuronal loss and astrocytic activation were ameliorated by pretreatment with WRPE.

## 4. Discussion

In the present study, we demonstrated that WRPE containing polyphenols and flavonoids exerted neuroprotective effects via antioxidative and anti-inflammatory activities in glutamate-treated NSCs and KA-challenged mice.

Generally, over-stimulation of glutamatergic neurons is major risk factor of excitotoxicity [29]. The mechanism of KA-induced seizure-mediated neuronal damage involves over-activation of glutamate receptors, which trigger excessive calcium influx into the neurons, eventually leading to necrotic and apoptotic neuronal deaths [1]. In our cresyl violet- and DAPI-stained findings, there was extensive injury and the loss of hippocampal pyramidal neurons, in addition to degenerative functional change in FJC staining, in the mice experienced severe epileptiform seizures.

Since oxidative stress plays a critical role in excitotoxicity [30], antioxidants such as resveratrol, vineatrol, and curcumin have been demonstrated to exhibit neuroprotective effects [7,8,29]. In our previous study, WRPE was found to have a potent antioxidant activity [31]. In the present study, the antioxidant potentials of WRPE were also confirmed via ABTS and DPPH radical-scavenging and lipid peroxidation assays. Furthermore, WRPE exerted a neuroprotective effect in human neural stem cells that were exposed to glutamate, a major excitotoxin in CNS.

It is well known that phenolic and flavonoid compounds are secondary metabolites with high antioxidative and anti-inflammatory properties [32,33]. The activities of phenolic compounds in vitro and *in vivo* are related to the number of hydroxyl functional groups in their structures [34]. As active ingredients that are responsible for the antioxidative and neuroprotective effects of WRPE, GC-MS analysis of the butanol fraction of WRPE revealed that it contained 2,3-dihydro-3,5-dihydroxy-6-methyl-4H-pyran-4-one (4.95%), 5-(hydroxymethyl)-2-furancarboxaldehyde (23.73%), and 1,2,3-benzenetriol (pyrogallol) (43.60%) composing 72.30% of the total peak area [24]. Pyrogallol is especially known as an antioxidant and anti-allergic component of WRPE. In addition, it was confirmed in the present study that an ethanolic (50%) extract of WRPE contain seven phenolic compounds including gallic acid, gentisic acid, (+)-catechinic acid, caffeic acid, veratric acid, hesperidin, and cinnamic acid. Based on these characteristics of ingredients, WRPE was inferred to have potent antioxidant and neuroprotective activities, which were confirmed in the ABTS- and DPPH-scavenging, lipid peroxidation, and glutamate-mediated cytotoxicity studies.

In our previous studies, WRPE has been shown to improve allergic dermatitis, skin aging, and especially ischemic stroke via its antioxidant and anti-inflammatory properties [21,22,23,24]. Therefore, we extended the activity of WRPE to excitotoxic brain injury, in which oxidative and inflammatory processes play an important role. KA injection induces epileptiform seizures, and enhances oxidative parameters in the blood and brain [13]. Similarly, in the present study, KA-induced seizures increased the brain TBARS level following diminished antioxidant enzymes (GPx1, PHGPx, SOD1, and SOD2), indicating that oxidative stress is involved in KA-induced brain damage, as previously suggested [35]. Notably, such seizures and alterations in antioxidant enzymes and TBARS were potently reversed by pretreatment with WRPE. These results suggest that WRPE can prevent the ROS-mediated detrimental effects that are caused by KA in the mouse brain.

Over the past decades, experimental and clinical findings have supported a crucial role of inflammatory processes in epilepsy, particularly in the mechanism underlying the generation of seizures [36]. Seizures induce high levels of inflammatory mediators such as IL-1β, TNF-α, TGF-β, and IL-6 in specific brain regions [37]. Upon excitotoxic brain injury, the prototypic inflammatory cytokines are up-regulated in activated microglia and astrocytes that trigger a cascade of downstream inflammatory events in neurons and endothelial cells of the blood-brain barrier (BBB) [30,36,38,39]. Strong IL-1β and IL-1R1 immunoreactivities were found in perivascular astrocytes and endothelial cells, which are associated albumin extravasation in the brain tissue reflecting BBB breakdown [40]. In turn, cytokines and other inflammatory mediators contribute to both excitotoxic and apoptotic neuronal deaths [41].

Interestingly, excitotoxicity also contributes to neuronal injury in diverse acute and chronic neurodegenerative diseases including cerebral infarction and traumatic brain injury (TBI) [42]. In particular, a rapid-onset inflammatory response is triggered in glia during seizures induced by chemo-convulsants [9]. The rapid release of HMGB1 from neurons, microglia, and astrocytes following epileptic injuries has been proposed as a crucial event for the initiation of brain inflammation and the reduction in the seizure threshold [43]. HMGB1 is considered a danger signal released from injured or stressed cells to alert the microenvironment of an immediate or ongoing injury. Its interaction with toll-like receptor 4 (TLR4) activates related inflammatory events [44]. Notably, NF-κB pathway is a prototypical signaling pathway governing the expression of proinflammatory genes including cytokines, chemokines, and adhesion molecules. As inferred from the anti-epileptic activity, down-regulation of HMGB1 and NF-κB expressions by administration of WRPE might inhibit the occurrence of seizures and subsequent inflammatory processes, in a consistent manner as also observed in NSCs.

Notably, iNOS is an enzyme that is responsible for production of nitric oxide (NO) mediating oxidative and inflammatory responses during epileptogenesis [45]. Astrocytes are the most abundant cells in the brain and serve as an important source of inflammatory mediators during courses of neuroinflammation [46]. Activation of the TNF-α-iNOS-NO pathway occurred in parallel with the increased number of activated astrocytes, which were confirmed by up-regulation of the production of GFAP. Inducible COX2, producing prostaglandins, is expressed in excitatory neurons and up-regulated in many organs by a wide variety of inflammatory stimuli, and especially, it plays an important role in seizure-induced leukocyte infiltration, astrogliosis, microglial activation, and breakdown of the BBB [47]. Moreover, COX2 is up-regulated by seizure activity and produce large amounts of PGs [48] that induce deleterious effects during epilepsy in collaboration with NO [49]. Activated astrocytes are important source of inflammatory COX2, in addition to iNOS [50]. Importantly, excitatory brain injury triggers the over-activation of astrocytes and, in turn, inflammatory process aggravates the brain damage: i.e., the increased expression of GFAP, an astrocyte cytoskeletal protein, is a quantitative marker of neural tissue injury [51]. Our present study revealed that the GFAP immunoreactivity as well as iNOS and COX2 expressions in the brain tissue and NSCs were markedly suppressed by treatment with WRPE, suggesting the pivotal role of its anti-inflammatory activity.

## 5. Conclusions

WRPE containing polyphenols and flavonoids exerted neuroprotective effects via antioxidative and anti-inflammatory activities. Specifically, WRPE exhibited radical-scavenging and lipid peroxidation-inhibitory activities in vitro, and protected against glutamate-induced cytotoxicity in neural stem cells. Also, pretreatment with WRPE attenuated epileptiform seizures in KA-challenged mice, and reversed hippocampal pyramidal cell loss and astrocyte activation, in which the antioxidant and anti-inflammatory parameters were restored. Therefore, it is suggested that WRPE could be a good candidate as an anti-epileptic or neuroprotective agent for clinical trials to attenuate seizure-related brain injury.

## Figures and Tables

**Table 1:** High-performance liquid chromatographic (HPLC) conditions for the analysis of phenolic acid.

**Instruments**	HPLC (ACME 9000 system, Younglin, Anyang, Korea)
**Column**	Mightysil RP-18GP column (5 μm, 4.6 × 250 mm)
**Mobile phase**	A (0.1% (*v*/*v*) acetic acid in acetonitrile), B (0.1% (*v*/*v*) acetic acid in water) 0 → 2 min (A:B = 92:8 → A:B = 90:10) 2 → 27 min (A:B = 90:10 → A:B = 70:30) 27 → 50 min (A:B = 70:30 → A:B = 10:90) 50 → 51 min (A:B = 10:90 → A:B = 0:100) 51 → 60 min (A:B = 0:100) 60 → 70 min (A:B = 0:100 → A:B = 92:8)
**Detector**	UV detector (280 nm)
**Flow rate**	1.0 mL/min
**Injection volume**	20 μL

**Table 2:** Sequences of the primers used in the current study.

**Gene Name**	**Accession No.**	**Human Primer**	
		**Forward (5′-3′)**	**Reverse (5′-3′)**
COX2	NM_000963	AAGTGCGATTGTACCCGGAC	GTGCACTGTGTTTGGAGTGG
GPx1	NM_000581	ACACCCAGATGAACGAGCTG	CAAACTGGTTGCACGGGAAG
IL6	NM_001318095	AGTGAGGAACAAGCCAGAGC	ATTTGTGGTTGGGTCAGGGG
iNOS	XM_011524860.2	ACAACAAATTCAGGTACGCTGTG	TCTGATCAATGTCATGAGCAAAGG
NF-kB	NM_001165412	GTGGTGCGGCTCATGTTTAC	CGTCTGATACCACGGGTTCC
PHGPx	NM_002085	CAGTGAGGCAAGACCGAAGT	CCGAACTGGTTACACGGGAA
SOD1	NM_000454	ATGACTTGGGCAAAGGTGGA	GGGCGATCCCAATTACACCA
SOD2	NM_000636	GGAGAACCCAAAGGGGAGTTG	GCCGTCAGCTTCTCCTTAAAC
TNF-α	NM_000594	GCTGCACTTTGGAGTGATCG	TCACTCGGGGTTCGAGAAGA
GAPDH	NM_001289746	AAGAAGGTGGTGAAGCAG	GTCAAAGGTGGAGGAGTG
**Gene Name**	**Accession No.**	**Mouse Primer**	
		**Forward (5′-3′)**	**Reverse (5′-3′)**
COX2	NM_011198	GAACCTGCAGTTTGCTGTGG	ACTCTGTTGTGCTCCCGAAG
IL-6	NM_031168.1	TCCAGTTGCCTTCTTGGGAC	AGTCTCCTCTCCGGACTTGT
iNOS	NM_010927.3	CTATGGCCGCTTTGATGTGC	TTGGGATGCTCCATGGTCAC
NF-kB	NM_008689	CACTGCTCAGGTCCACTGTC	CTGTCACTATCCCGGAGTTCA
SOD2	NM_013671	GGAGCAAGGTCGCTTACAGA	GTGCTCCCACACGTCAATC
TNF-α	NM_013693	TACCTTGTTGCCTCCTCTT	GTCACCAAATCAGCGTTATTAAG
GAPDH	NM_008084	CGTGCCGCCTGGAGAAACC	TGGAAGAGTGGGAGTTGCTGTTG

**Table 3:** List of antibodies used in the current study.

Epitope	Company	Cat. Number	Dilution	2° Ab (IgG)
HMGB1	Cell Signaling	#3935	1:1000	anti-rabbit
TGFβ	Santa Cruz	sc-146	1:200	anti-rabbit
iNOS	Santa Cruz	sc-651	1:200	anti-rabbit
COX2	Santa Cruz	sc-1746	1:500	anti-goat
GFAP	Santa Cruz	sc-51908	1:200	anti-mouse
GAPDH	Cell Signaling	#2118	1:1000	anti-rabbit
